# Novel Wavelet Real Time Analysis of Neurovascular Coupling in Neonatal Encephalopathy

**DOI:** 10.1038/srep45958

**Published:** 2017-04-10

**Authors:** Lina F. Chalak, Fenghua Tian, Beverley Adams-Huet, Diana Vasil, Abbot Laptook, Takashi Tarumi, Rong Zhang

**Affiliations:** 1 Department of Pediatrics, UT Southwestern Medical Center, Dallas, TX, USA; 2 Department of Bioengineering, University of Texas at Arlington, Arlington, TX, USA; 3 Department of Clinical sciences Statistics, UT Southwestern Medical Center, Dallas, TX, USA; 4 Brown University, Providence, RI, USA; 5 Department of internal medicine, University of Texas Southwestern Medical Center, Dallas, TX, USA

## Abstract

Birth asphyxia constitutes a major global public health burden for millions of infants, despite hypothermia therapy. There is a critical need for real time surrogate markers of therapeutic success, to aid in patient selection and/or modification of interventions in neonatal encephalopathy (NE). This is a proof of concept study aiming to quantify neurovascular coupling (NVC) using wavelet analysis of the dynamic coherence between amplitude-integrated electroencephalography (aEEG) and near-infrared spectroscopy in NE. NVC coupling is assessed by a wavelet metric estimation of percent time of coherence between NIRS S_ct_O_2_ and aEEG for 78 hours after birth. An abnormal outcome was predefined by a Bayley III score <85 by 18–24 m. We observed high coherence, intact NVC, between the oscillations of S_ct_O_2_ and aEEG in the frequency range of 0.00025–0.001 Hz in the non-encephalopathic newborns. NVC coherence was significantly decreased in encephalopathic newborns who were cooled vs. non-encephalopathic controls (median IQR 3[2–9] vs.36 [33–39]; p < 0.01), and was significantly lower in those with abnormal 24 months outcomes relative to those with normal outcomes (median IQR 2[1–3] vs 28[19–26], p = 0.04). Wavelet coherence analysis of neurovascular coupling in NE may identify infants at risk for abnormal outcomes.

Despite hypothermia therapy for hypoxic neonatal encephalopathy (NE), 50% of treated newborns have disabilities at 12–18 months of age^1^,^2^. The asphyxia insult impairs fetal cerebral blood flow (CBF) and is manifested postnatally by a distinctive neonatal encephalopathy (NE), which is usually classified after birth using the clinical modified Sarnat stages as mild, moderate and severe NE^3^. Recent reports suggest new evidence of cognitive impairment in a subset of 30–50% of “mild NE” cases, who are currently not recognized or offered any therapies^4^,^5^. While adjuvant therapies are being sought, an important focus of research is to recognize which neonates are in need of therapies to improve outcome^6^,^7^. The challenge in the field of neonatal brain injury has been the difficulty to clinically discern the mild-moderate NE severity within the short therapeutic window after birth. A sensitive and specific physiological marker that directly assesses neurovascular function in real-time, and is a marker of clinical outcomes, is critically needed to guide therapies^8^,^9^,^10^.

The neurovascular unit consists of neurons, astrocytes, endothelial cells of blood–brain barrier (BBB), myocytes, pericytes and extracellular matrix components which play an important role in the delivery of oxygen and nutrients to the brain and the maintenance of cerebral circulation homeostasis^9^,^11^,^12^. Individual metrics of electroencephalography^6^,^13^,^14^,^15^ and near-infrared spectroscopy (NIRS)^16^,^17^ have been used independently to assess the function of neurovascular unit, but the ability to study coupling in real time of NIRS and EEG in HIE has received little attention^11^,^17^,^18^,^19^ These methods demonstrated that a severe asphyxia insult leads to impaired function, but the extent of dysfunctional neurovascular coupling (NVC), and its impact on clinical outcome of newborns with encephalopathy cannot be currently assessed in real time due to limited methodology^20^,^21^.

Intrinsic oscillating activities at multiple time scales have been described in cortical neurons across mammalian species^22^. These oscillations span from the very low to ultra-fast frequencies and although difficult to measure, are functionally relevant, phylogenetically conserved across species, and play important roles in facilitating synaptic plasticity and brain functional connectivity^22^. Cerebral blood flow (CBF) and brain tissue oxygenation also manifest oscillations at multiple time scales which occur simultaneously with changes in neuronal activities suggesting NVC^23^.

Our previous work demonstrated a new time-frequency domain wavelet analysis of the dynamic relationship between spontaneous oscillations in mean arterial pressure and NIRS-measured cerebral tissue oxygen saturation (S_ct_O_2_) allowed quantification of cerebral autoregulation at the bedside in newborns with encephalopathy^24^,^25^. We use the novel wavelet analysis to quantify NVC at the bedside, as defined in a global context to reflect the relationship between each of 1) NIRS S_ct_O_2_, a surrogate of CBF under steady state conditions, and 2) the amplitude integrated EEG (aEEG), which measures ensemble neuronal activity. The aims in this proof of concept study are to assess the feasibility of using the wavelet analysis to quantify NVC at the bedside, and its potential to stratify NE severity and identify newborns at high risks for abnormal outcomes following neuroprotective therapies.

## Results

### Patient characteristics

Twenty inborn newborns with fetal metabolic acidosis were evaluated and screened during the one year study period. Of those, ten newborns had simultaneous aEEG and NIRS recordings which were suitable for wavelet analysis. The study was approved by UT Southwestern IRB and parental consent was obtained. These ten newborns comprised eight with moderate to severe encephalopathy who received hypothermia and two with no encephalopathy who did not meet criteria for cooling and served as references. Patient characteristics are summarized in Table 1. Neuromonitoring was initiated at 12 ± 2 hours for duration of 60 ± 6 hours with no artifacts during recording. No scalp edema or any other complications were noted and no leads were replaced during the duration of the recording. No infant had seizures during the recording period. Only one infant had clinical seizures within 6 hours of life prior to enrollment. All cooled infants were categorized to have moderate encephalopathy, except one who was severe. All infants with a normal outcome had continuous patterns on aEEG. Sleep wake cycles (SWCs) were identified by aEEG in eight infants, occurring every 60–90 minute as summarized in Table 1, two infants showed no SWC. MRI was available on all patients and was performed at a median age of 7 days. The MRI was abnormal in 4 neonates with white matter injury and watershed infarcts, one had added basal ganglia involvement. Abnormal outcome with Bayley III scores <85 at 18–24 months occurred in six of the ten infants and performance in each domain is summarized in Table 1.

### Wavelet findings

**Figure 1:**
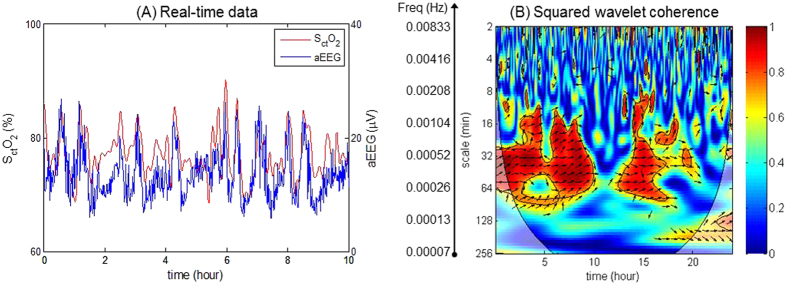
Wavelet-based S_ct_O_2_-aEEG coherence showing intact neurovascular coupling (NVC). This extract is from a cooled neonate with normal outcome (MRI showed no injury and Bayley III >85. (**A**) An enlarged segment of the real-time S_ct_O_2_ and aEEG data. (**B**) Squared wavelet coherence, 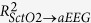, where the x-axis represents time, the y-axis represents scale in minute representing the range of frequencies, and the color scale represents the magnitude of *R*^2^. Significant coherence between the S_ct_O_2_ and aEEG is seen in a very low-frequency (VLF) range of 0.00025–0.001 Hz.

**Figure 2:**
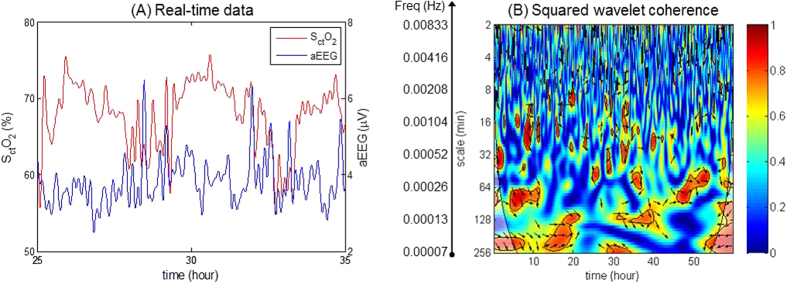
Wavelet-based S_ct_O_2_-aEEG coherence showing low neurovascular coupling. This infant had white matter injury on MRI. The Bayley III scores at 18 months are: cognitive 71, language 70, and motor 85. (**A**) An enlarged segment of the real-time S_ct_O_2_ and aEEG data. (**B**) Squared wavelet coherence, 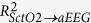, where the x-axis represents time, the y-axis represents scale of frequencies. No significant areas of coherence are seen through the range of time and frequencies studied.

Significant in-phase coherence (p < 0.05) between S_ct_O_2_ and aEEG was identified by the wavelet analysis in the two non-cooled references, in the wavelet scale of 16–64 minutes equivalent to a very low-frequency (VLF) range of 0.00025–0.001 Hz. Similar findings were observed in the cooled infants with a normal Bayley outcomes at 18–24 months post hypothermia therapy (Fig. 1). In contrast, wavelet-based S_ct_O_2_→aEEG coherence showed decreased NVC for cooled infants with abnormal outcomes. Figure 2, highlights a case example of low NVC where the infant subsequently had white matter injury on MRI day 7 of life, and a Bayley III cognitive score of 70 at 18 months of age. Of note, the observed frequency of NVC coherences was outside the range of SWC frequencies summarized in Table 1, and occurred even in the two cases where no SWC was observed, hence the analysis of NVC did not appear to be influenced by the sleep-awake cycles.

**Figure 3:**
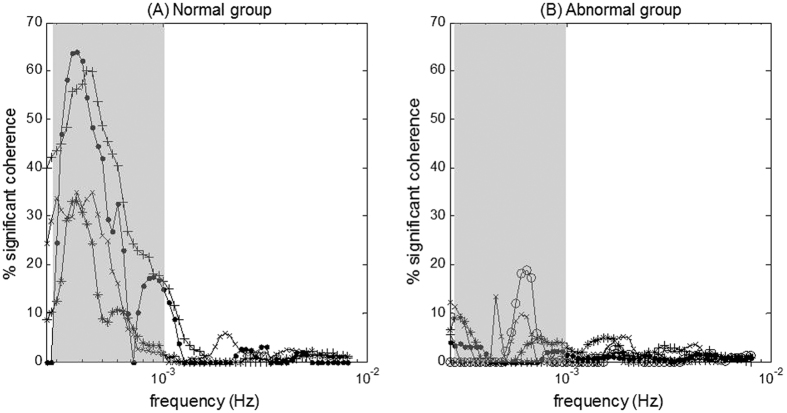
Individual data of significant S_ct_O_2_→aEEG in-phase coherence from newborns quantified in: (**A**) normal outcome group (n = 4), and (**B**) abnormal outcome group (n = 6). The two non-cooled are labeled by + and • in the left panel. Significant differences between normal vs. abnormal groups (*p* < 0.05) was observed in the frequency range of 0.00025–0.001 Hz, highlighted by gray shade.

**Figure 4:**
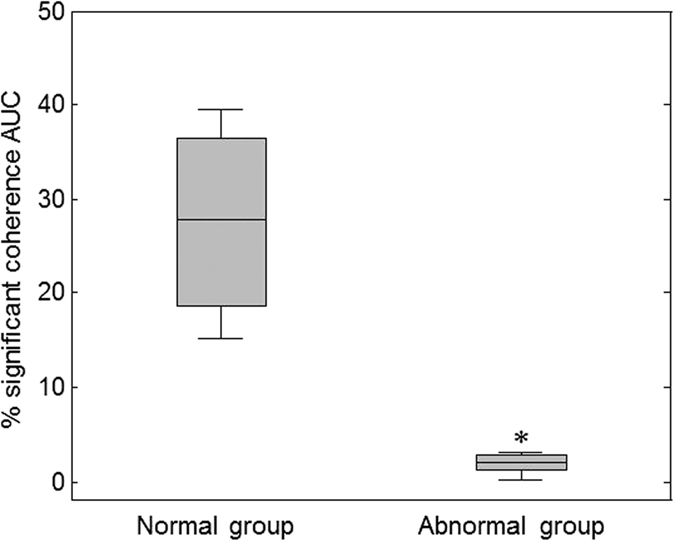
Area under the curve (AUC) of significant SctO2-aEEG coherence in infants categorized by the 18–24 developmental outcomes. X axis shows infants divided into two groups: normal (n = 4) and abnormal outcomes (n = 6) based on Bayley <85 at 18–24 months. Y axis is AUC for significant NVC coherences. Boxplot (median, 25% and 75% percentiles) for the % NVC coherence AUC over the frequency range of 0.00025–0.001 Hz, p = 0.01 by Exact Wilcoxon Rank Sum test.

Figure 3 Describes the percentage of significant coherence plotted for each individual patient with or without abnormal outcomes at 18–24 months. According to this figure, the most distinct differences between infants with normal vs. abnormal outcomes were observed in a very low-frequency (VLF) range of 0.00025–0.001 Hz (wavelet scale *s* = 16–64 minutes). The boxplot distribution Fig. 4 shows significant differences in NVC coherence between groups. NVC coherence was significantly lower in newborns with abnormal outcomes compared to normal outcomes (median IQR 2[1–3] vs 28[19–26], p = 0.04).

## Discussion

This proof of concept study demonstrated the feasibility of using wavelet analysis of the dynamic coherence function between S_ct_O_2_ and aEEG to assess NVC at the bedside in asphyxiated newborns with encephalopathy. The preliminary findings suggest that wavelet measures of NVC in the first 72 hours of life are associated with long-term outcomes following NE, with and without hypothermia therapy.

The studies of spontaneous oscillations has always been entwined with neuronal network functionality and self-organization^26^. Understanding the physiological mechanisms of self-emerging oscillations of brain neuronal activity not only provide insight into brain function, but also may assist in the recognition of newborns with HIE who need added therapies to hypothermia. In the normal brain at term, a tight temporal and spatial coupling exists between neuronal activation and CBF^27^. Coupling between NIRS-measured S_ct_O_2_ and EEG activity ensures a rapid spatial/temporal increase in the CBF in response to neuronal activation under normal conditions^28^. The capillary endothelial cells, astrocytes and neurons, together forming a neurovascular unit, are involved in the tightly coupled regional blood flow in response to local metabolic demands^29^. The underlying physiological mechanisms of NVC are complex, but hyperemic responses appear to be mediated by astrocytes^30^,^31^,^32^, occurring within seconds of localized brain activity. The resulting increased local CBF is likely to be larger than the concomitant oxygen consumption, resulting in increased NIRS saturation^33^.

The prevailing concept of NVC, introduced over a century ago by Roy and Sherrington^34^, has been applied mostly in the modern-day neuroimaging studies of brain function with positron emission tomography (PET) and functional MRI in adult subject^35^. However, these techniques are either invasive and therefore cannot be applicable in the fragile newborn, or susceptible to movement artifact and therefore do not permit a real time analysis of continuous dynamics. Moreover, none of these methods can measure the multi-frequency aspect of neurovascular coupling. Oscillatory coupling of neuronal networks using NIRS and EEG has only been described prior in a cohort of healthy newborns^28^. The latter study involved non-sick preterm newborns and established coupling via intermittent recordings of integrated EEG and NIRS during normal gestational growth^28^. However, it is not known how these observations apply in the normally developing newborn apply to sick newborns with NE.

The new findings of significant coherence between the oscillations in S_ct_O_2_ and aEEG (in the VLF range of 0.00025–0.001 Hz) suggest the presence of intact NVC in large time scales in this group of term newborns with NE and normal outcomes. These slow rhythms have also been shown in adults to synchronize large spatial domains affecting connectivity, repair and functions^36^, and the present study findings would suggest they are also likely to be impaired with NE associated with abnormal outcomes.

The wavelet analysis of NVC in this study may provide new insights into the regulation of cerebral hemodynamics and neuronal function. The sophisticated analysis is not limited to a specific monitoring device, but can be adapted to a multitude of non-invasive devices (such as continuous BP monitors, NIRS, and aEEG) that are all available at the bedside. While high NIRS cerebral saturation and suppressed aEEG background neuronal activity have been each separately linked to abnormal outcomes in HIE^17^,^19^, prior studies have provided only description of such uncoupling, without quantifiable metrics. The wavelet coherence analysis of NIRS-aEEG signals integrates the diagnostic information obtained from each device individually to permit continuous evaluation of coupled cortical activity and perfusion^37^,^38^,^39^. This study demonstrated that the large time-scale functional oscillations of the neurovascular unit can be measured non-invasively and continuously in asphyxiated newborns. The findings are novel, as the dynamic coherence relationship between changes in NIRS signal and neuronal activity has not been previously established in hypoxic NE or during hypothermia using non-invasive devices at the bedside.

Study strengths include (1) a prospective design, (2) a real time wavelet analysis of continuous recordings over long time durations which allowed quantification of a wide range of VLF oscillations, and (3) use of standardized long term developmental outcomes in in newborns with encephalopathy. The study ensured maintaining an optimal temperature at 33.5 °C, steady state conditions, and absence of high impedance or artifacts during the wavelet recording.

This proof of concept study is limited by a small number of patients, allowing only exploratory analyses. The NVC monitoring of the parietal channel might be limited in providing information regarding deep structures like the basal ganglia^40^, but was selected as it is representative of the global injury covering the watershed white matter that is usually seen in HIE.

In conclusion, the study demonstrated the feasibility of using the novel wavelet coherence analysis of spontaneous oscillations in S_ct_O_2_ and aEEG to quantify the NVC in newborns with encephalopathy. The new wavelet bundle approach, once validated in large NE cohorts promises to significantly impact the early NE stratification and prediction of neurocognitive outcomes. The ability to prospectively monitor the global neurovascular unit functions in real time, via novel approaches as delineated in this study, could in the future provide a paradigm shift to the field of neonatal neuro-critical care and improve the selection of candidates in neuroprotection studies.

## Methods

### Subjects

This study included inborn infants ≥36 weeks of gestation and birth weight ≥1800 grams who were admitted to the neonatal intensive care unit (NICU) at Parkland Hospital of Dallas and had perinatal asphyxia with metabolic acidosis and a clinical exam showing encephalopathy in the first six hours from June 2012 to June 2013. Exclusion criteria included the presence of congenital anomalies, imminent death, or transfer to another facility. The study was approved by the Institutional Review Board of the University of Texas Southwestern Medical Center. Informed consent was obtained from both parents before enrollment. All methods were performed in accordance with the relevant STROBE guidelines and regulations and all patient data is de-identified.

Perinatal acidemia was determined by measuring blood gases in umbilical arterial blood that is obtained from a double clamped section of umbilical cord at birth. Specifically, criteria were as defined by the National Institute of Child Health and Human Development (NICHD) Neonatal Research Network study of whole body hypothermia^41^. A neurological examination was performed by one study investigator (LC) within 6 hours of birth using the NICHD classification for modified Sarnat staging to establish the severity of encephalopath^42^.

The reference comprised newborns with fetal acidosis screened by a detailed neurological examination, who had no abnormalities and did not meet the criteria to receive hypothermia. The hypothermia group was comprised of newborns with a composite exam consistent with moderate or severe encephalopathy who did receive hypothermia therapy. Encephalopathy was defined as the presence of either moderate or severe signs in at least 3 of the following 6 categories as per NICHD cooling trials: (1) level of consciousness (moderate is lethargic, severe is stupor or coma), (2) spontaneous activity (moderate is decreased activity, severe is no activity), (3) posture (moderate is distal flexion or complete extension, severe is decerebrate), (4) tone (moderate is hypotonia, severe is flaccid), (5) primitive reflexes (moderate is a weak suck [or incomplete Moro reflex], severe is an absent suck [or absent Moro reflex]), and (6) autonomic nervous system^41^,^43^. Whole body hypothermia was started within 6 hours after birth and achieved by placing the newborn on a cooling blanket (Blanketrol II, Cincinnati Sub-Zero) and maintaining the esophageal temperature at 33.5 °C by the blanket servomechanism for 72 hours.

### Monitoring

#### Amplitude Integrated EEG (aEEG)

Electrical brain activity was monitored by using an amplitude-integrated EEG monitor (Natus Medical, San Carlos, CA, USA). Five hydrogel electrodes were placed by the same research personnel (DV) according to the international 10–20 system predefined criteria: ground, P3, C3, C4 and P4. The skin was prepared using Neo-Prep gel, and the impedance was monitored during the study for the analysis to include only recordings with impedance <5 kΩ. Electro cortical activity was classified according to a standardized classification as (1)Continuous: Continuous activity with lower (minimum) amplitude around 5 to 10 mcV and maximum amplitude of 10 to 25–50) mcV; (2)Discontinuous: Discontinuous background with minimum amplitude below 5 mcV, and maximum amplitude above 1 mcV; (3) Burst-suppression: Discontinuous background with minimum amplitude without variability at 0 to 1 mcV and bursts with amplitude >25 mcV; (4) Low voltage: Continuous background pattern of very low voltage (below 5 mcV); (5) Inactive, flat: Primarily inactive (isoelectric below 5 mcV)^44^.

The EEG signals as per aEEG manufacturing standards were amplified and bandpass-filtered (1 to 50 Hz) to minimize artifacts and electrical interferences, rectified, and then peak to peak amplitudes were measured and smoothed using a moving average of 0.5 seconds^45^. The resulting upper margin amplitude, lower margin amplitude and bandwidth (BW) for the selected P3–P4 channel were quantitatively calculated and displayed using Brainz Analyze Research (v1.5) software (Natus Medical). In addition, cyclical pattern typically occurring about every 60 to 90 minutes indicating sleep wake cycles (SWC) with broadened band width (discontinuous activity) representing quiet sleep and narrow band width (continuous activity) representing wakefulness or active sleep were checked and highlighted.

#### Near-Infrared Spectroscopy (NIRS)

The INVOS (Somanetics) spatially-resolved NIRS oximeter was used (INVOS 4100–5100; Somanetics, Troy, MI). A neonatal probe (neonatal, SomaSensor) containing a light-emitting diode and two distant sensors was placed on the infant’s head, in close proximity to the P3 and P4 aEEG leads. The sampling rate of NIRS signal was every 30 seconds.

#### Data pre-processing

The S_ct_O_2_ and aEEG were synchronously recorded every minute with a Vital Sync™ system (Somanetics Corporation, Troy, Michigan). Both the S_ct_O_2_ and aEEG data were first inspected to identify artifacts, which were removed by linear interpolation between neighboring data points, followed by a second-order polynomial de-trending to remove the slow drifts of each time series.

#### Wavelet coherence analysis

Wavelet transform coherence is a time-frequency domain analysis, which characterizes the cross-correlation between two time series at multiple time scales and over time^46^. Details of this method were recently published^47^ and a brief description is provided in the online supplement. The concept is to use a time varying, squared cross-wavelet coherence, *R*^2^, between two time series in multiple time scales without a priori assumptions of linearity and stationarity^48^. *R*^2^ ranges between 0 and 1 and can be conceptualized as a localized correlation coefficient in the time-frequency domain between any two pre specified variables. The two variables selected for evaluation of NVC were the NIRS S_ct_O_2_ and aEEG in this study. The statistical significance of the estimated coherence between the two times series against noise background was assessed based on theMonte Carlo method^49^. The wavelet coherence metrics include the relative phase, Δ*φ*, and the squared wavelet coherence, *R*^2^, in the interrogated range of frequencies (*f*_*wt*_ = 0.97/*s*; *s* represents wavelet scale in seconds) and over the time of the recorded S_ct_O_2_ and aEEG durations. NVC is represented as the significant in-phase coherence (Δ*φ* ≈ 0) between the S_ct_O_2_ and aEEG oscillations. Therefore to quantify the results, we first select a phase range of interest. Within the selected phase range, the percentage of significant coherence, *P(s*), is quantified as the percentage of time during which the S_ct_O_2_→aEEG coherence is statistically significant from the noise background (*p* < 0.05) by using the Monte Carlo simulation. *P(s*) is a function of wavelet scale, *s*, or Fourier frequency, *f*_*wt*_.

### Neurodevelopmental outcomes

Brain MRI was performed on day 7–10 using conventional T1- and T2-weighted spin echo sequences to evaluate for injuries in the basal ganglia, cortical or watershed areas, by using the NICHD classification^50^. Neurological examination and psychometric testing were conducted 18 to 24 months of age based on the Bayley Scales of Infant Development III (Bayley III for short) in three domains: cognitive, language and motor^1^,^51^. All study infants completed follow up including the non-cooled controls. An abnormal outcome was predefined by abnormal MRI scores >1 and Bayley III scores <85 in any of the cognitive, language or motor domains. Examiners of neurodevelopmental outcomes were blinded to the aEEG and NIRS results.

### Statistical analysis

This Wavelet method generates a large ensemble of surrogate data set pairs with the same power spectrum as the input datasets. Wavelet coherence is estimated for each pair. Then the coherence of input datasets is statistically tested against those of surrogate datasets with a null hypothesis that the signal is generated by a stationary noise background. The statistically significant squared cross-wavelet coherence, *R*^*2*^, against noise background and the corresponding phase between the time series of S_ct_O_2_ and aEEG were determined, and the percentage of time durations during which *R*^2^ was statistically significant (*p* < 0.05) was calculated relative to the whole recording period for each infant. Exact Wilcoxon Rank Sum test were used to compare the wavelet coherence (% of significant coherence) across NE severity in cooled infants (moderate-severe NE combined since only 1 severe NE) vs. controls as well as between the infants with normal outcome vs. abnormal outcome groups. This included between-group comparisons for each wavelet scale at a significance of *p* < 0.05, and a comparison of area under the curve (AUC) over the selected frequency range at a significance of *p* < 0.01. For this pilot study, multiple comparison corrections were not applied.

## Additional Information

**How to cite this article:** Chalak, L. F. *et al*. Novel Wavelet Real Time Analysis of Neurovascular Coupling in Neonatal Encephalopathy. *Sci. Rep.* **7**, 45958; doi: 10.1038/srep45958 (2017).

**Publisher's note:** Springer Nature remains neutral with regard to jurisdictional claims in published maps and institutional affiliations.

## Supplementary Material

Supplementary Information

## Figures and Tables

**Table 1:** Clinical Characteristics.

Gestation Age (weeks)	Birth Weight (Kg)	Sarnat Stage	Sex	Gas Base Deficit	aEEG Pattern	aEEG SWC (cycle/hour)	aEEG Bandwidth (μV)	NIRS S_ct_O_2_ (Mean)	MRI	Bayley III at 18–24 m
^38	3.3	1	F	19	1	0.6	11	75	0	105-99-95
**^**37	2.5	1	M	21	1	1	13	76	0	90-97-107
41	3.5	2	M	16	1	0.8	8	76	0	90-95-97
37	3.0	2	M	17	1	0.7	6	80	0	95-98-105
38	3.5	2	F	17	2	None	7	89	0	65-95-82
37	2.9	2	M	20	1	0.5	10	90	(1a)	80-70-90
38	3.1	2	M	22	2	1	9	86	(1b)	71-70-85
38	2.8	3	F	16	1	0.7	11	82	(1b)	90-75-97
38	4.7	2	M	19	2	0.5	9	70	0	70-85-85
36	2.6	2	F	30	3	None	9	88	(2)	65-80-85

^Denotes the 2 controls who did not receive hypothermia. Clinical severity of encephalopathy using modified Sarnat staging listed as follows: 1, no encephalopathy, 2: moderate NE, 3: severe NE (in a minimum of 3/6 categories). Infants highlighted in grey had the abnormal outcomes. aEEG score 1: continuous, 2, Discontinuous, 3, Burst Suppression. SWC sleep wake cycle. MRI NICHD scores: 0 = no injury; 1a = minimal cerebral lesions only and no area of watershed infarction; 1b = more extensive cerebral lesions, 2 = basal involvement or watershed infarction noted without any other cerebral lesions, 2 = involvement of basal ganglia or infarction and additional cerebral lesions. Bayley outcomes for cognitive-language-motor scores are listed sequentially.
