# Supplementary material for: Novel Wavelet Real Time Analysis of Neurovascular Coupling in Neonatal Encephalopathy
Source: Sci Rep. 2017 Apr 10;7:45958. doi: 10.1038/srep45958 (PMC5385531; doi:10.1038/srep45958)
Supplement: Supplementary Information [file srep45958-s1.pdf]

Title Novel Wavelet Real Time Analysis of Neurovascular Coupling in Neonatal Encephalopathy

Lina F Chalak, MD, MSCS; Fenghua Tian, Ph.D.; Beverly Huet MS, Diana Vasil RNC, Abbot Laptook, MD, Takashi Tarumi, Ph.D. and Rong Zhang, Ph.D.

Supplement:

Detailed wavelet coherence analysis

Wavelet coherence is based on continuous wavelet transform of time series in the time-frequency domain by successively convolving the time series with the scaled and translated versions of a mother wavelet function ψ_0. The wavelet transform of a time series $x(n)$ of length N, which is sampled from a continuous signal at a time step of Δt, is defined as:

$$W^X(n,s) = \sqrt{\frac{\Delta t}{s}} \sum_{n'=1}^{N} x(n)\, \psi_0^*[(n'-n)(\frac{\Delta t}{s})] \quad (1)$$

where n is the time index, s denotes the wavelet scale that is in inverse proportion to frequency, and * indicates the complex conjugate. The mother wavelet employed in this study is a Morlet wavelet (with ω_0 = 6), which provides a good trade-off to capture both the time and frequency characteristics of the observed $S_{ct}O_2$ and aEEG signals. By using the Morlet wavelet, the relationship between the wavelet scale and its corresponding Fourier frequency (f_{wt}) is $f_{wt} = 0.97/s$.

The cross-wavelet transform of two time series, $x(n)$ and $y(n)$, is defined as:

$$W^{XY}(n,s) = W^X(n,s) W^{Y^*}(n,s) \quad (2)$$

where the modulus $|W^{XY}(n,s)|$ represents the joint power of $x(n)$ and $y(n)$, and the complex argument $\Delta\varphi(n,s) = tan^{-1}\{\frac{Im[W^{XY}(n,s)]}{Re[W^{XY}(n,s)]}\}$ represents the relative phase between $x(n)$ and $y(n)$.

In analogy to the magnitude-squared coherence (MSC) function based on Fourier transform (Zhang et al., 1998), a squared cross-wavelet coherence $R^2(n, s)$ is defined as (Torrence and Webster, 1999):

(3)

where S is a smoothing operator in the time-frequency (scale) domain which uses a weighted running average in both the time and scale directions. $R^2(n, s)$ ranges between 0 and 1 and can be conceptualized as a localized correlation coefficient between $x(n)$ and $y(n)$ in the time-frequency domain. To test the statistical significance of $R^2(n, s)$ against noised background, a Monte Carlo method is implemented. Briefly, this method generates an ensemble of surrogate data pairs (n = 300) that have the same model

coefficients as the real time series data based on a first-order autoregressive (AR1) model. Wavelet coherence is calculated for all of the surrogate data pairs. Then the significance level of $R^2(n, s)$ of the observed time series is determined by comparing with those from the surrogate data at each time and wavelet scale. In this study a 95% confidence interval ($p < 0.05$) is used for statistical testing.
